# Variation of Immune Cell Responses in Humans Reveals Sex-Specific Coordinated Signaling Across Cell Types

**DOI:** 10.3389/fimmu.2022.867016

**Published:** 2022-03-28

**Authors:** Gabriela K. Fragiadakis, Zachary B. Bjornson-Hooper, Deepthi Madhireddy, Karen Sachs, Han Chen, David R. McIlwain, Matthew H. Spitzer, Sean C. Bendall, Garry P. Nolan

**Affiliations:** ^1^ Department of Microbiology & Immunology, Stanford University, Stanford, CA, United States; ^2^ Department of Medicine, Division of Rheumatology, University of California San Francisco, San Francisco, CA, United States; ^3^ CoLabs, University of California San Francisco, San Francisco, CA, United States; ^4^ Bakar ImmunoX Initiative, University of California San Francisco, San Francisco, CA, United States; ^5^ Immunology Program, Stanford University, Stanford, CA, United States; ^6^ Department of Otolaryngology – Head and Neck Surgery, University of California, San Francisco, San Francisco, CA, United States; ^7^ Department of Microbiology & Immunology, University of California, San Francisco, San Francisco, CA, United States; ^8^ Parker Institute for Cancer Immunotherapy, San Francisco, CA, United States; ^9^ Chan Zuckerberg Biohub, San Francisco, CA, United States; ^10^ Department of Pathology, Stanford University, Stanford, CA, United States

**Keywords:** mass cytometry, CyTOF, immune cells, signaling, humans

## Abstract

Assessing the health and competence of the immune system is central to evaluating vaccination responses, autoimmune conditions, cancer prognosis, and treatment. With an increasing number of studies examining immune dysregulation, there is a growing need for a curated reference of variation in immune parameters in healthy individuals. We used mass cytometry (CyTOF) to profile blood from 86 humans in response to 15 *ex vivo* immune stimuli. We present reference ranges for cell-specific immune markers and highlight differences that appear across sex and age. We identified modules of immune features that suggest there exists an underlying structure to the immune system based on signaling pathway responses across cell types. We observed increased MAPK signaling in inflammatory pathways in innate immune cells and greater overall coordination of immune cell responses in females. In contrast, males exhibited stronger pSTAT1 and pTBK1 responses. These reference data are publicly available as a resource for immune profiling studies.

## Introduction

Immune function is critical for effective vaccine responsiveness, wound healing, and protection against infection, autoimmunity, and cancer (1). Although the ability to measure elements of the immune response has improved dramatically with advances in gene expression analysis, cytokine profiling, and cytometry (2–4), clinical assessment relies predominantly on the comparatively simple complete blood count as the indicator of immune health (Mayo Clinic). This disparity between our current technical capabilities and the methods employed in healthcare offers the potential for dramatic improvements in assessments of immune health.

Mass cytometry has demonstrated great potential as an immune monitoring tool (5–7). The ability to measure over forty proteins per single cell enables deep profiling of the immune system from a patient’s blood or tissue sample, yielding information regarding the phenotype as well as the behavior of cells, such as signaling activity (2). These and other studies have leveraged mass cytometry to assess immune differences in individuals in different clinical contexts (5, 6, 8–10), but a reference for healthy human immune variation at steady-state is lacking.

As part of the Cross-Species Immune Atlas, we looked to create a reference of human immune variation that can serve as a baseline in immunological studies, like efforts made in genomics and other fields (11), and one that is complementary to single-cell mapping initiatives across tissues such as the Human Cell Atlas (https://www.humancellatlas.org/). We also looked to expand our understanding of immune cell phenotype and signaling responses in humans and animal model species commonly used in drug development to identify which of these features are conserved across species. In the data presented here and in the accompanying paper by Bjornson-Hooper et al. (12), we profiled blood in response to 15 immune stimuli (including cytokines, growth factors, and microbial products) from 86 human subjects, 88 non-human primates (rhesus macaques, cynomolgus macaques, African green monkeys), and 50 mice using a 39-parameter mass cytometry immune profiling panel. Through the standardization of immune stimuli and antibody panels across the five species and the minimization of technical error using automation and single lots of reagents, we produce a publicly available and curated reference with complete documentation for other researchers.

Analysis of this dataset provides a set of reference ranges for a large set of measured immune parameters, such as immune cell frequencies and functional responses, and reveals coordinated sets of immune features that were grouped into modules based on correlated variation as measured across individuals. These immune modules suggest an immune system structure of coordinated signaling capacity across cell types and enabled stratification of our donors by sex. While reference ranges for most features overlap between sexes, we found that females had stronger inter- and intra-module correlation than males, suggesting a greater degree of immune cell signaling coordination in females and an increased signaling capacity in inflammatory signaling pathways in innate immune cells. Such differences in immune responses between sexes may inform the immune bases for observed differences in pathology such as infection susceptibility and autoimmune syndrome prevalence.

## Results

### Detailed Immune Profiling of Healthy Individuals Provides a Window into Immune State

In order to curate a high-quality reference of the human immune system, we sought to heavily control the performance of assays on a large set of donors and reduce technical variation during the processing of samples. A cell phenotyping antibody panel capable of delineating major immune populations and an intracellular antibody panel measuring cell signaling in response to a set of clinical and disease-relevant stimuli were developed in conjunction (5, 9, 13, 14) (**Table S1**). To minimize error and technical variability, antibodies targeting 23 surface antigens and 16 intracellular signaling proteins were metal-conjugated in bulk and lyophilized into single-use antibody cocktails that were stable for long-term storage and curtailed variation due to small volume pipetting and antibody degradation (see **Methods**).

**Figure 1:**
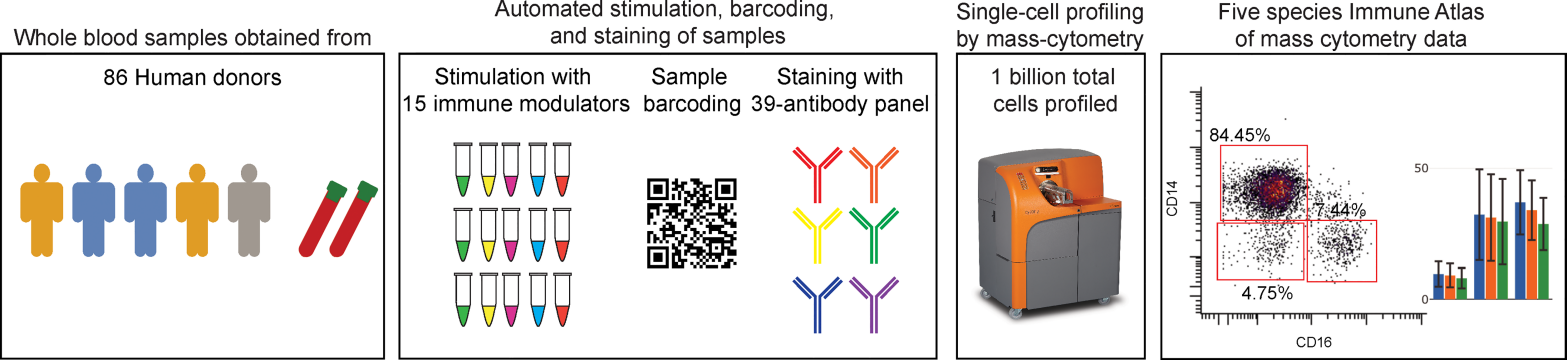
Detailed immune profiling of healthy individuals using mass cytometry. Generation of immune reference dataset. Whole blood samples were taken from 86 human donors and divided in 16 aliquots. Each aliquot was stimulated with a different cytokine, microbial agent, or left untreated. Samples were barcoded and stained with a 39-parameter antibody panel, and cells were analyzed by mass cytometry. The curated data is available as a public resource on https://flowrepository.org (accession FR-FCM-Z2ZY).

Samples were collected from 86 healthy humans along with self-reported demographics including age, height, weight, and sex (**Table S2**). Note: Gender was not included in the questionnaire and this study, therefore, examines results related to self-reported sex rather than gender. Fresh whole blood samples were used to eliminate variation and inconsistencies associated with PBMC isolation protocols. Samples from each volunteer were collected in a single vial and then divided into 16 aliquots that were stimulated with 15 immune modulators, including cytokines, growth factors, cell type-specific agonists, and microbial antigens, or left unstimulated (**Figure 1** and **Table S3**). All stimulation conditions from a single donor were barcoded using mass-tags and combined into a single tube per donor to ensure consistent staining volume and processing. Samples were stimulated, fixed, lysed, barcoded, and stained automatically through a robotics platform, and prior to every sample run, CyTOF quality controls tests were performed. All samples from an individual were run in the same batch. Samples were resuspended with normalization beads that facilitated control of variation across all CyTOF runs as previously described (15).

This resulted in a reference dataset available for use in future immune profiling studies that contains baseline cell abundances and signaling levels obtained from 86 healthy individuals, their immune responses following 15 stimulation conditions and with minimal technical variation. For the focus of this investigation, 12 major immune populations were gated (although the larger number of surface markers present in the panel enables identification of further cell types), and their responses to various stimulation conditions were explored. The high-dimensional nature of mass cytometry measurements combined with the large stimulation panel yielded 2,160 immune “features” from the data set. We then isolated 199 features that are known stimuli-activated signaling pathways in cell types relevant in major biological responses for further analysis (**Figure S2**, **Methods**).

### Immune Variation Enables Detection of Response Modules Predominantly Defined by Signaling Proteins

**Figure 2:**
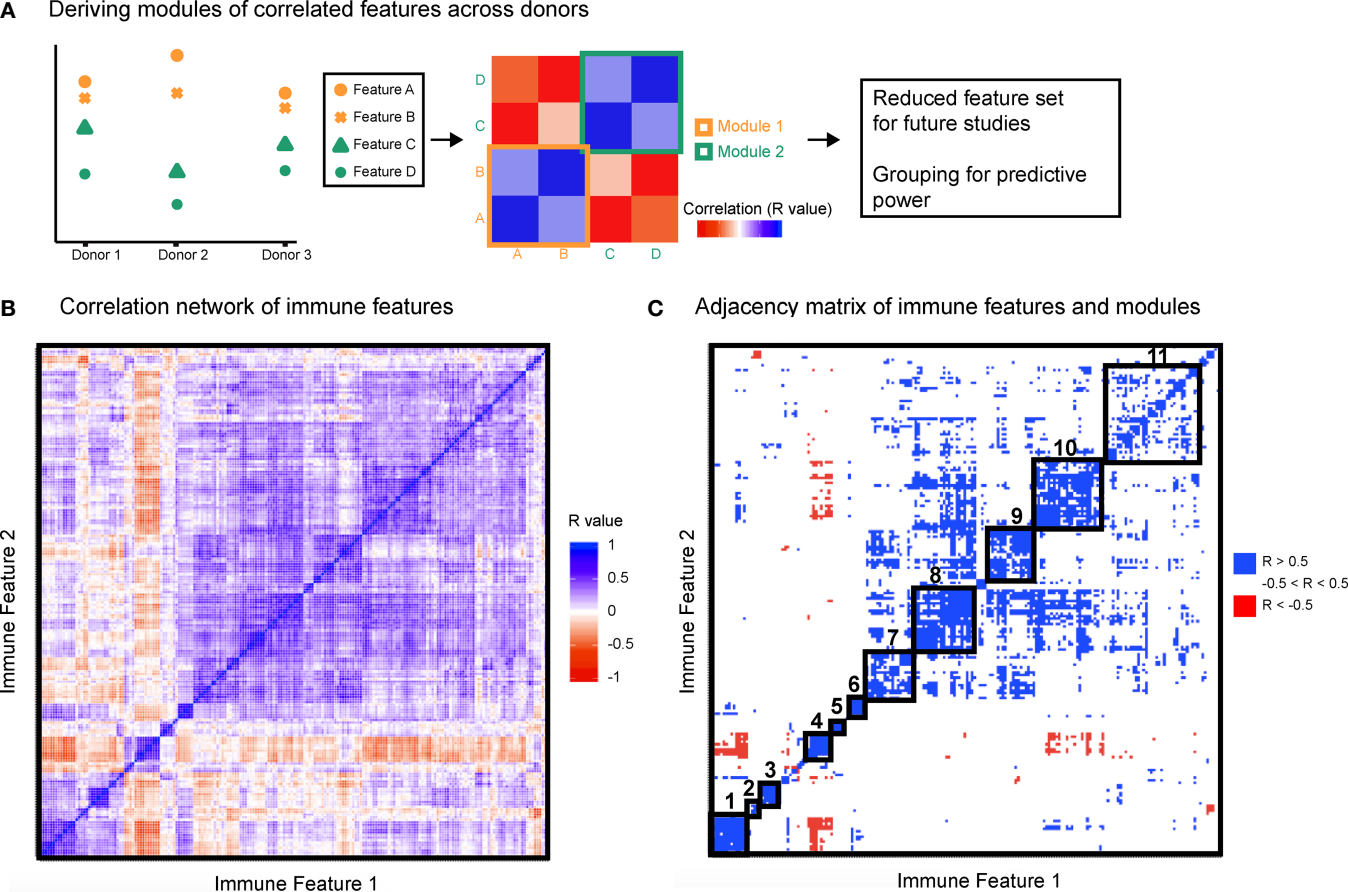
Immune variation enables detection of response modules predominantly defined by signaling proteins. All features were correlated with one another across donors. **(A)** Highly correlated features were identified and annotated as groups of correlated features, or modules. **(B)** Clustered correlation heat map of the 199 features. Immune features were clustered on each axis based on similarity of correlation coefficient (R-value). Heat map is colored by R-value. **(C)** R-values were binned yielding an adjacency matrix. R-values from -1 to -0.5 are red, R-values from -0.5 to 0.5 are white, and R-values from 0.5 to 1 are blue. Modules were drawn based on visualized groups of highly correlated features (black boxes).

To leverage the multi-parameter profiling of the donors we used each individual in the cohort as an observed instance of a genetic or environmental perturbation of the immune state. Each feature was correlated with every other feature across donors to produce a correlation map (**Figures 2A, B**) and hierarchically clustered to place features that were correlated near one another. After stringently thresholding for features that were strongly correlated to one another (|R| > 0.5), the resulting adjacency matrix helped elucidate the presence of 11 modules (**Table S4**), most of which were characterized by signaling protein, rather than by cell type or stimulation condition (within-module correlation mean R = 0.68 versus correlation between all features mean R = 0.20, **Figure 2C**). The signaling modules (1, 2, 4, 6, 7, 8, and 10) suggest a level of immune regulation and structure based on signaling pathway activity that is present across many cell types within an individual. The consistency of signaling capacity across conditions suggested that the *ex vivo* stimulations probed an intrinsic regulation of each signaling pathway across an individual’s cells.

This grouping revealed that if an individual had lower phosphorylated levels of a given signaling protein in each cell type and condition compared to the population average, that individual was likely to also have lower levels of that phosphorylated signaling protein across other cell types and conditions (**Table 1**). The consistency of signaling capacity across conditions suggested that the *ex vivo* stimulations probed an intrinsic regulation of each signaling pathway across an individual’s cells.

**Table 1:** Attributes of features contained within each module.

Module	Proteins	Cell types	Conditions
1	pSTAT1	CD8 T cells, CD4 T cells, DCs, NK cells, B cells	IL-6, IFNa, IFNb, IFNg
2	pSTAT1	Basophils, monocytes	PMA/iono, IFNa, IFNb
3	pERK1/2, pCREB, pMAPKAPK2	CD4 T cells, CD8 T cells, B cells	PMA/iono
4	IkB	B cells, NK cells, CD4, CD8, monocytes, DCs	CD40L, TNFa, LPS, R848
5	pP38, pERK1/2	Monocytes, basophils	Anthrax
6	pSTAT1	CD14 monocytes, CD16 monocytes	IFNa, IFNb, IFNg
7	pCREB, pP38, pERK1/2	Monocytes, neutrophils, basophils	GMCSF, IL-6, R848, LPS, PMA/iono
8	pSTAT5, pSTAT6	Monocytes, neutrophils, DCs, T cells	IL-2, GMCSF, IFNa, IFNb
9	pTBK1, pCREB, pMAPKAPK2, pP38, pERK1/2	DCs, NK cells, monocytes	TNF
10	pSTAT4, pSTAT5, pSTAT6	CD8, CD4, DCs, NK cells, neutrophils, monocytes	IFNa, IFNb, IL-4, IL-6
11	pTBK1, pCREB, pMAPKAPK2, pP38, pERK1/2	Monocytes, DCs, neutrophils	PMA/iono, LPS, R848, GMCSF

For the immune features classified in each module, the proteins, cell types, and stimulation conditions common to the majority of features are listed. The most prevalent type of attribute for a given module is highlighted in blue.

In contrast to the signaling pathway dominant modules, there was no clear organization of modules by specific cell lineages (**Table S4**). Whereas lymphoid and myeloid cells tended to be grouped together, several cell types shared highly correlated activity of a particular signaling pathway. Similarly, although many of the stimuli used are pleiotropic and activated several signaling pathways, the organization of only three modules (3, 5, and 9) were driven by stimuli (**Table S4**). These results demonstrate that the activation of different signaling pathways elicited by a particular stimulus is not as coordinated as the activity of a given signaling pathway to different stimuli. Therefore, the immune set point in healthy individuals reveals the coordination of signaling pathway activity, which defines each person’s propensity to respond to numerous immunological stimuli.

At a practical level, these results also imply the evaluation of a smaller number of conditions may provide nearly equally meaningful information (i.e., a surrogate) with respect to the general immune state. Therefore, with this dataset as a foundation, future studies using more focused diagnostic immune monitoring can be performed using a subset of proteins and conditions as a traditional flow cytometry assay (**Table S5**). This is especially important in the clinical setting, where typically only flow cytometry is readily available. Surrogate markers for most modules are present in this subset of proteins and conditions and their normalized values were highly correlated with derived module scores, the normalized average of the immune features within each module (**Figure S3**, **Methods**).

### Immune Structure Stratifies Immune Responses Between Males and Females

**Figure 3:**
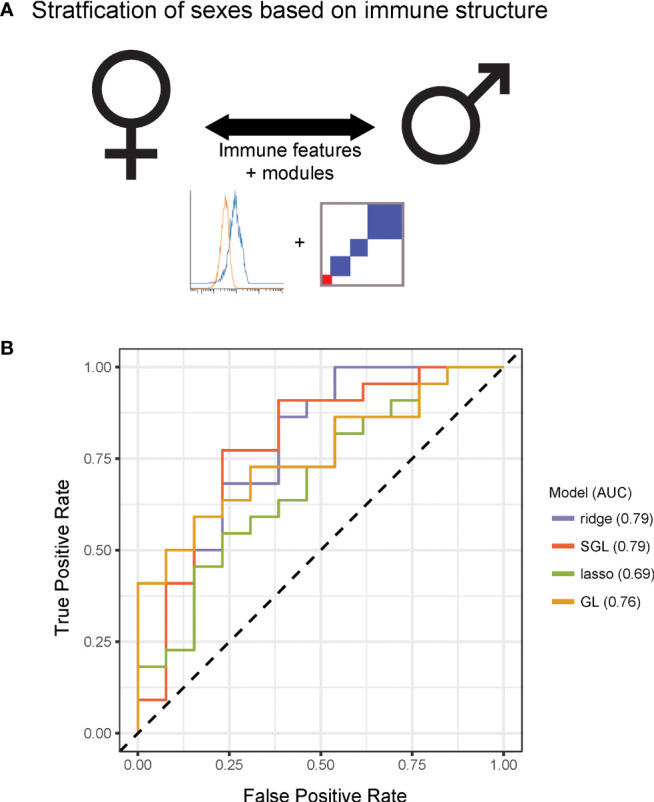
Immune modules enable improved stratification of immune responses across sex and age. **(A)** Schematic of modeling approach for sex differences. **(B)** Receiver operating characteristic curve for ridge, lasso, group lasso, and sparse group lasso for prediction of sex based on immune structure. Performances on test data were as follows [stated as percent correct classification, area under the curve (AUC)]: ridge, 68%, 0.79; lasso, 60%, 0.69; group lasso, 60%, 0.76; sparse group lasso, 74%, 0.79.

Having observed that measured immune responses were organized into signaling-based modules, we next sought to determine whether this organization could characterize differences in immune state between individuals. We were first interested in whether this modular structure enabled stratification by sex (**Figure 3A**). We performed predictive modeling, including models that allowed for input of the module assignments as a means of incorporating the higher order relationships from the data. The data were randomly split into a training set of 50 donors and a test set of 36 donors. Four models were optimized using the training data including two regularized methods that did not use module groupings [ridge (16) and lasso (17)] and two that used the module groupings (group lasso (18) and sparse group lasso (19), (see **Methods**). We assessed the predictive performance of each model on the test data, and found that the immune data significantly correctly classified the data by sex, the sparse group lasso model performing the best (74% correct classification on test; AUC 0.79) (**Figure 3B**). Notably, the model that used the internal structure of the data in addition to the immune features best defined differences in immune state between males and females.

The lasso model selected immune features that included signaling proteins pTBK1 and pSTAT1 (positive coefficients, higher in males) and features that included pERK1/2, pP38, and pCREB (negative coefficients, higher in females, **Table S6**; see further discussion of selected features later in the manuscript). The sparse group lasso model was more inclusive and selected 35 features from modules 1, 3, 4, 5, 7, 8, 10, and 11, that included the majority of measured signaling proteins (**Table S6**). To assess how much predictive information could be captured on the coarse-grain module level, we also trained a lasso model on personalized module scores rather than on raw immune features. The model selected module scores 1, 5, and 7, and had a classification rate of 68% on the test set (AUC 0.67). This model outperformed the lasso model and performed comparably well to our ridge model, revealing that this simplified module-based model was able to harness much of the predictive information for classification by sex (**Table S6**). Taken together, these modeling results suggest that the modular structure we observed may capture relevant variability in immune state, thus revealing sex differences in immune signaling.

### Females Have Increased Coordination of Immune Cell Signaling Capacity

**Figure 4:**
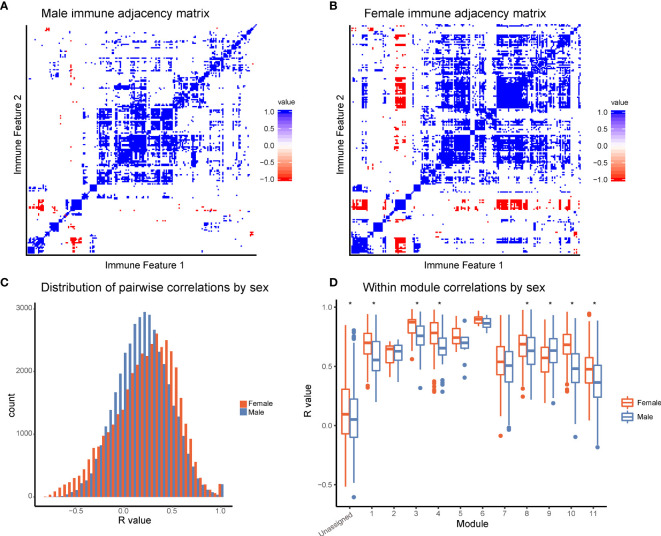
Females have increased coordination of immune cell signaling capacity. **(A)** Adjacency matrices of 199 immune features across male donors. R-values were binned; R-values from -1 to -0.5 are red, from -0.5 to 0.5 are white, and from 0.5 to 1 are blue. Feature order was set by the clustering order on the full dataset. **(B)** Adjacency matrices of 199 immune features across female donors. R-values were binned; R-values from -1 to -0.5 are red, from -0.5 to 0.5 are white, and from 0.5 to 1 are blue. Feature order was set by the clustering order on the full dataset. **(C)** Distribution of correlation coefficients (R-values) of each pairwise feature in male donors (turquoise) compared to female donors (pink). Distributions were significantly different (p-value = 2.2x10-16, Wilcoxon sum-rank test). **(D)** Box-plots of correlation coefficients (R-values) within modules grouped by sex. Modules are numbered as in **Figure 2C**. Females (pink) had higher levels of correlation in modules 1, 3, 4, 8, 10, 11, and unassigned (Wilcoxon sum-rank test, adjusted p-value <.05). Males (turquoise) had higher levels of correlation in module 9. (* indicates Wilcoxon sum-rank test adjusted p-value <.05).

To further explore differences in immune structure between male and female donors in our cohort, immune feature correlations were calculated separately for each sex. From the correlation networks, adjacency matrices were produced that maintained the feature order and module grouping from the full data set (**Figures 4A, B**). This analysis revealed that features from female donors were more highly correlated with one another than features were within male donors (**Figure 4C**, p-value = 2.2x10^-16^). In seven modules, mean within-module correlation was higher in females than in males, whereas only one module had higher mean correlation in males and females (**Figure 4D**). This suggests a higher degree of immune regulation and coordination among components of the female immune response, versus the male immune response in this dataset.

### Females and Males Have Distinct Immune Response Profiles

**Figure 5:**
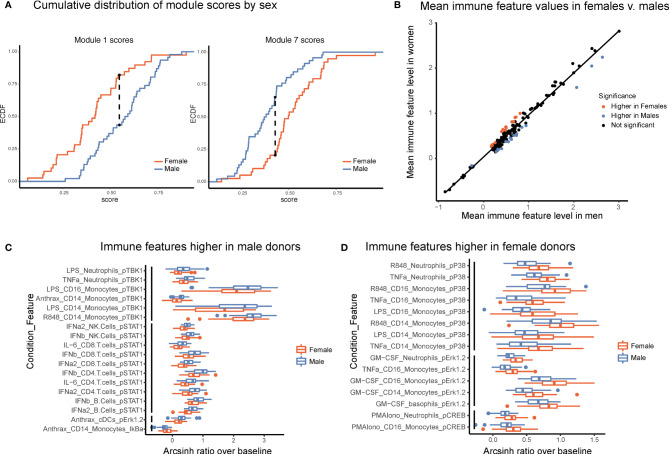
Males and females have distinct immune response profiles. **(A)** Modules with significantly different module scores between males and females. Empirical cumulative distribution function (ECDF) shown for modules scores from module 1 (left) and module 7 (right). Dashed line shows the maximum distance between distributions. Significance determined by Kolmogorov-Smirnov test, adjusted p-value <.05. **(B)** Mean levels of each feature in samples from female donors versus male donors. Red indicates significantly higher in females; blue, significantly higher in males; and black, not significantly different based on an FDR < 1% (SAM unpaired). **(C)** Boxplots of the features significantly higher in males (FDR < 1%, SAM unpaired). Features are grouped by signaling protein (black bars). Decreased IkBa plotted with male donors to reflect increased degradation of IkBa leading to higher levels of p-NFkB. **(D)** Boxplots of the features significantly higher in females (FDR < 1%, SAM unpaired). Features grouped by signaling protein (black bars).

Several studies have suggested that there are intrinsic immune differences between males and females. Females have drastically higher incidence of autoimmune disease, whereas males have poorer tolerance of infection and responses to vaccines (20, 21). These clinical differences, as well as interactions between sex-linked genes, hormones, and the immune system (22), strongly suggest that differences exist between the male and female immune systems. Module scores, calculated as the average of an individual’s normalized levels of each immune feature in a module, were significantly different between males and females in two modules (**Figure 5A**, Kolmogorov-Smirinov test). The module higher in males (module 1) was predominantly made up of pSTAT1 features across several lymphocyte subsets from interferon and IL-6 conditions, whereas the module higher in females (module 7) was comprised of pCREB, pP38, and pERK1/2 in innate immune cell types (**Table 1**). Interestingly, module 1 scores did not correlate with module 7 scores within a given sex, suggesting that on a per-individual basis having a stronger sex-based phenotype in one module does not have implications for the sex-based phenotype in the other module (s1). Other trends in module-based sex differences that did not pass our significance threshold when correcting for multiple hypothesis testing included modules 3 and 5 (higher in males) and modules 4 and 9 (higher in females) (**Figure S5**).

To further explore this result, we performed an exhaustive assessment of all thresholder immune features in the dataset. Whereas feature medians were globally highly similar between the sexes (R = 0.985), a subset of features was significantly different between male and female donors (SAM, FDR < 0.01, **Figure 5B**). Differences were highly consistent by signaling protein. Of the 18 features that were significantly higher in male donors, six were pTBK1 features and 10 were pSTAT1 features across conditions and cell types (**Figure 5C**). Similarly, of the 15 features significantly higher in female donors, all were features involving pP38, pCREB, or pERK1/2 (**Figure 5D**). These were the same proteins that were selected by our lasso model of sex-differences. Interestingly, responses to TNFa, LPS, and R848 were higher in males in certain cell-type specific proteins and higher in females in others, suggesting that the response to the same stimulus can be differentially regulated across signaling pathways. In contrast, all significantly different responses to GM-CSF were exclusively higher in females than in males, whereas responses to interferons, IL-6, and *Bacillus anthracis* antigen were exclusively higher in men. In addition, not all pathways responsive to a given stimulus were different between sexes; for example, whereas GM-CSF stimulates both a pERK and a pSTAT5 response in monocytes, only the response to pERK was higher in females. Notably, all of the features higher in female donors were from inflammatory cell types and signaling pathways known to be involved in inflammation, consistent with higher rates of autoimmunity and enhanced responses to infection observed in females.

## Discussion

The goals of this analysis were 1) to generate a set of mass cytometry data collected from healthy human subjects, curated with explicit immune features to be used as a reference in immune monitoring studies and 2) to leverage the multi-parameter measurements of healthy immune variation to gain insights into immune organization. This highly controlled dataset was acquired with strict processes to minimize technical variation and consists of immune measurements on samples from 86 individuals in response to 15 different immune modulators and an unstimulated control. Analysis of 16 signaling protein responses in nine canonical cell types resulted in 2,160 feature measurements per individual, filtered to 199 features that met our threshold of responsiveness. The raw data, as well as the curated and filtered immune features, have been made available to the research community as a public database for future reference in immune monitoring studies at https://flowrepository.org (accession FR-FCM-Z2ZY).

Using each feature as a standalone measurement does not utilize the data to its full potential, as this high-dimensional dataset contains information about how the immune system is preconfigured to respond in a coordinated manner to immune stimuli. To extend the analysis beyond univariate measurements toward an understanding of the relationship between features, we leveraged the multi-parameter measurements available wherein individuals were used as instances of genetic or environmental perturbation. The merits of this type of approach have been shown with the identification of co-regulated features and module detection in gene expression data (23) and in cytometry data in specific immune modulatory contexts (13). Here we identified modules of correlated immune features across humans as a group, and males or females as sub-cohorts. The attribute common across features within a module was a shared signaling protein. This result reveals that 1) the immune system is structured in such a way that the signaling propensity of a given pathway in an individual is coordinated across cell types and across different immune perturbations and 2) that there is a degree of redundant information contained in these measurements such that future studies may perform smaller assays that can be readily performed in the clinical setting. An example of a restricted set of parameters that could be used for representative immune monitoring using traditional flow cytometry is provided in **Table S5**.

In our predictive models, the elucidated immune structure informed stratification by individual self-reported sex. Incorporating module assignments from this work, which used not only the features themselves but also the relationships between immune features, enabled the detection of differences in immune responses, demonstrating that the detected internal structure captures feature relationships that vary within human demographic groups. The modular structure improves prediction and also distills the resulting models into interpretable results. This in turn suggests that this framework may be useful in analyses of infection susceptibility, autoimmunity burden, and treatment response.

The use of mass cytometry for immune profiling allowed for the examination of cell-specific signaling pathway responses in addition to cell frequencies, and analysis of this dataset allowed for detailed characterization of a broad set of differences between the male and female immune systems. Significantly higher responses in male donors than in females were predominantly detected in pSTAT1 in lymphocytes and pTBK1 in myeloid subsets, whereas female donors had higher levels of pERK1/2, pCREB, and pP38 features in monocytes and neutrophils (**Figure 5**). The female immune system has been shown to have higher inflammatory phenotypes than the male immune system both clinically and at the cellular level (24). Notably, this includes increased interferon responsiveness and innate immune pathway activation in macrophages from females (25), which is consistent with our findings of increased MAP-kinase signaling in innate immune inflammatory cells in females compared to males. In contrast, neutrophils and monocytes were more abundant in males than in females (**Figure S6**). Perhaps this disparity in cell abundance partially compensates for inflammatory pathways within monocytes and neutrophils having a greater response capacity in females. Interestingly, males have worse outcomes in a multitude of microbial infections (21, 26, 27) and it was therefore surprising that pSTAT1 and pTBK1 capacity was higher in males than females. This could be a compensatory mechanism to counter the fact that males have lower interferon alpha production in response to inflammatory stimuli and therefore require more sensitive signaling responses (28).

Our analysis also opened a window into more nuanced aspects of sex-specific immune regulation. Females had a greater degree of correlation of responses across immune features than males, which may point to greater coordination of immune responses and structure. It is unclear why immune cell signaling responses in males are more independent of one another, although it may reflect a more defined immune set point in females. Future work will be required to examine the nature of this sex-specific coordination.

Although immune structure varies by demographic parameters such as sex, it remains unclear how this variation affects immune pathology. Future prospective studies may be warranted to determine how immune set points relate to risk of development of future immune pathology. It will also be informative to explore whether demographic variations in higher-order interactions between immune features highlighted in this study are maintained or exaggerated in the context of disease. Future work should be aimed at incorporating data from longitudinal draws from the same donor and integrating different complementary sources of immune information, such as gene expression and cytokine analysis. Longitudinal blood draws from the same donor would provide an insight into how stable the immune state of a healthy donor is and its consistent response to an immune modulator that could provide an insight as to whether there is an optimal time for the introduction of a perturbation, such as an immunization, or a time when the individual is susceptible to infection. Incorporation of information from other immunological assays would help provide an even richer picture of the immune state. This work, and the accompanying manuscript by Bjornson-Hooper et al. (12), provide the first analyses of the Cross-Species Immune Atlas dataset. We hope this curated resource as well as these initial analyses will enable future human immune monitoring studies as well as more rational pre-clinical studies.

## Methods

The methods for reagent development and sample processing are those used for the Cross-Species Immune Atlas project and are redundantly stated here and in Bjornson-Hooper et al. (12).

### Data Generation

#### Stimuli

Reagents used for stimulations are listed in **Table S3**. Stimuli were tested in whole blood over a range of concentrations to select the optimal working concentration. All reagents were diluted such that the same volume of each achieved the desired level of stimulation. Stimulation reagents were then aliquoted into single-use plates and stored at -80°C with the exception of those indicated in **Table S3**, which were dispensed at time of use due to storage requirements. All cytokines were tested for endotoxin by the LAL method and verified to contain an amount less than that detectable by our phospho-flow assays (approximately 10 pg/ml). The LPS used was prepared by phenol-water extraction and contained small amounts of other bacterial components that activate TLR2. Gamma-inactivated vegetative *Bacillus anthracis* Ames (ANG-BACI008-VE) was obtained from the Department of Defense Critical Reagents Program through the NIH Biodefense and Emerging Infections Research Resources Repository, NIAID, NIH.

#### Blood

Venous human blood was obtained from the Stanford Blood Center and from All Cells (exempt, non-human subjects research) and from volunteers from the Stanford community under an IRB-approved protocol. Donor demographics, including age, height, weight, and sex, were self-reported by individuals through a survey.

#### Antibodies

Purified antibodies were purchased and conjugated in-house using DVS/Fluidigim MaxPar X8 metal conjugation kits. All antibodies were titrated for optimal signal-to-noise ratio, which was confirmed in at least two different individuals per species (three humans, two cynomolgus macaques, two rhesus macaques, three mice). All conjugations and titrations were well-documented, and records are available in the experiment data repository. Antibodies were lyophilized into LyoSpheres by BioLyph with excipient B144 as 4x cocktails. CyTOF antibody LyoSpheres were stress-tested for over one year and found to have no significant change in staining (not shown).

#### Stimulation and Staining

Stimulations and staining were carried out on an automation platform consisting of an Agilent Bravo pipetting robot, Agilent BenchBot robotic arm, Peak KiNeDx robotic arm, Thermo Cytomat C2 incubator, BioTek ELx405-UVSD aspirator/dispenser, BioTek MultiFlo FX four-reagent dispenser, Q.Instruments microplate shakers, Velocity11 VSpin centrifuges, and a custom chilling system contained in a negative-pressure biosafety enclosure. The VWorks robotic programs and log files are available upon request.

Whole blood was stimulated by mixing with a stimulus reagent and incubating in a humidified 37°C, 5% CO_2_ incubator for 15 minutes. Blood was fixed for 10 minutes at room temperature with 1.6% paraformaldehyde (PFA, Electron Microscopy Sciences) and lysed with 0.1% Triton-X100 in PBS for 30 minutes at room temperature (29). Cells were washed twice with PBS, then each of the 16 conditions for each donor was barcoded as previously described (30). Briefly, cells were slightly permeabilized with 0.02% saponin, then stained with unique combinations of functionalized, stable palladium isotopes. Each stimulation plate contained samples from 6 donors stimulated under 16 conditions. After stimulation, each plate was reduced to 6 wells, each containing the 16 conditions for one donor. Cells were washed once with staining media (CSM: 0.2% BSA in PBS with 0.02% sodium azide), blocked with human TruStain FcX block (Biolegend) for 10 minutes at room temperature with shaking, then stained with rehydrated extracellular LyoSpheres for 30 minutes at room temperature with shaking in a final volume of 240 µl. Cells were washed once with CSM, then permeabilized in >90% methanol at 4°C for 20 minutes. Cells were washed four times, then stained with intracellular lyospheres for 60 minutes at room temperature with shaking. Cells were washed once, then placed into 1.6% PFA and 0.1 µM natural iridium intercalator (Fluidigm) in PBS at 4°C until acquisition on a CyTOF. With few exceptions, cells were acquired within seven days of staining. From prior validation experiments, this amount of time imparted no significant effect on staining.

#### Acquisition

Prior to analysis on the CyTOF, cells were washed twice with water. Samples were acquired on a single DVS/Fluidigm CyTOF 2 fitted with a Super Sampler sample introduction system (Victorian Airship & Scientific Apparatus). Machine QC reports were run on the CyTOF between every barcoded set. Prior to data acquisition, the instrument was demonstrated to have Tb159 dual counts greater than 1,000,000 and oxidation less than 3%; if the instrument failed those criteria, it was cleaned, tuned or repaired as necessary. Approximately 4,800,000 events were acquired per sample.

#### Data Processing

##### Data Normalization, Debarcoding, and Manual Gating

Data were normalized and debarcoded using the data normalization software (15) and the single cell debarcoder tool described in (31) described previously. Data were gated and analyzed with https://cellengine.com/. To empower future studies, staining and gating were performed in keeping the cross-species universal mass cytometry staining and gating strategy described in Bjornson-Hooper et al. (12).

##### Derivation of Immune Features

Immune features were defined as follows. A standard data transformation was applied (transformed value = arcsinh(value/5)), and then the median values of each signaling protein (n = 15) were extracted per gated cell type (n = 9) for each condition. The medians from the unstimulated condition were subtracted from the medians from each stimulation condition (n = 16). The resulting value was defined as the immune feature measurement. This derivation resulted in 2,160 immune features (16*9*15).

In the interest of removing features that were not a result of a response to the stimulus, a threshold was set empirically at a mean immune feature value of 0.2. This threshold was chosen by looking at T cell responses versus monocyte responses to LPS (T cells do not have the LPS receptor, whereas monocytes do).

#### Statistical Modeling

##### Sex Differences in Immune Features

Significance of immune features between sexes was assessed using Significance Analysis of Microarrays (SAM) (32) using a false discovery rate of less than 1%. Analysis was performed in R using the same library. Sex differences in module scores were assessed using the Kolmogorov-Smirinov test, with a Benjamini-Hochberg correction for the twelve tests performed (11 modules + unassigned).

##### Regression Models

Models predicting donor sex based on immune features were optimized and evaluated using the R libraries glmnet for lasso, ridge, and elastic net; SGL for sparse group lasso; and grplasso for group lasso. The training set was initially sampled at random and kept consistent throughout for each outcome variable. Model performance was assessed on the samples not selected for the training set. For the elastic net analysis, the alpha parameter was tuned using k-fold cross-validation on three separate iterations of assigning k. This revealed that ridge (alpha = 0) had the lowest cross-validation error in each iteration (**Figure S7**). Code used for this study can be accessed through the Github repository at: https://github.com/gfragiadakis/FDA-library.

##### Module Derivation, Visualizations, and Scores

Modules were drawn and visualized, with the ability to extract feature groupings, using an R interface (in Shiny) written for this project. Remaining visualizations shown were produced in R using the ggplot2 library. Module scores were calculated based on the average of the normalized values of the features in each module.

## Data Availability Statement

The datasets presented in this study can be found in online repositories. The names of the repository/repositories and accession number(s) can be found below: Flowrepository, Accession number FR-FCM-Z2ZY, https://flowrepository.org/id/FR-FCM-Z2ZY.

## Ethics Statement

The studies involving human participants were reviewed and approved by Stanford Institutional Review Board. The patients/participants provided their written informed consent to participate in this study.

## Author Contributions

GF wrote the manuscript, contributed to data generation, conceived of and performed the data analysis. ZB generated the data and edited the manuscript. KS advised data analysis and edited the manuscript. HC and DRM contributed to data analysis and edited the manuscript. DM contributed to data generation and reagent optimization and edited the manuscript. MS contributed to data generation, advised data analysis, and edited the manuscript. SB provided advising and edited the manuscript. GN advised the study and edited the manuscript. All authors contributed to the article and approved the submitted version.

## Funding

The research discussed in this article was supported in part by the U.S. Food and Drug Administration (Contract No. HHSF223201210194C). GF was supported by the Stanford Bio-X graduate research fellowship and NIH grant T32GM007276. ZB was supported by NIH grant T32GM007276. MS was supported by NIH grant DP5OD023056. Additional support was provided by NIH awards 5R01CA18496804, 5R25CA18099304, 1R01GM10983604, 5UH2AR06767603, 1R01NS08953304 and R01HL120724 and FDA contract HHSF223201610018C.

## Author Disclaimer

This article reflects the views of the authors and should not be construed to represent the U.S. Food and Drug Administration’s views or policies.

## Conflict of Interest

ZB is involved in the commercial development of the platform used to host the data presented in this manuscript.

The remaining authors declare that the research was conducted in the absence of any commercial or financial relationships that could be construed as a potential conflict of interest.

## Publisher’s Note

All claims expressed in this article are solely those of the authors and do not necessarily represent those of their affiliated organizations, or those of the publisher, the editors and the reviewers. Any product that may be evaluated in this article, or claim that may be made by its manufacturer, is not guaranteed or endorsed by the publisher.

## Supplementary Material

Supplementary Figure 1 Manual gating strategy for all data. Gating strategy identifying 12 unique cell populations for one representative donor. Populations used for analysis are shown in red.

Supplementary Figure 2 Boxplot of reference ranges of 199 immune features that exceeded a mean value of 0.2 arcsinh ratio. Immune features are grouped by cell type and colored by condition.

Supplementary Figure 3 Surrogate marker performance. Comparison of individual’s surrogate markers from a reduced feature set relative to their module scores using the full data set. Value on the y-axis is the value of the immune feature normalized to the range of that feature. Spearman rank correlations are as follows for modules 1, 4, 6, 7, 8, 9, 10: 0.90, 0.91, 0.94, 0.63, 0.88, 0.83, 0.74, respectively.

Supplementary Figure 4 Module 1 scores do not correlate with module 7 scores within the sexes.

Supplementary Figure 5 Module score comparison between sexes. Comparison of module scores between sexes. Distributions marked with ** are significantly different when correcting for multiple hypothesis testing (Kolmogorov-Smirinov test, adjusted p-value < 0.05, h = 12). To show trends, all are modules are shown, those marked with * are significant without correcting for multiple hypothesis testing (Kolmogorov-Smirinov test, unadjusted p-value < 0.05).

Supplementary Figure 6 Sex differences in frequency data. CD14+ monocyte frequencies (of mononuclear cells) and neutrophil frequencies (of singlets) in samples from male and female donors. Frequencies are higher in samples from male donors (Significance analysis of microarrays, FDR <.01).

Supplementary Figure 7 Tuning alpha parameter for elastic net. Selection of alpha for elastic net modeling of sex differences. Three iterations of assigning k-groups for cross-validation are shown. Error is lowest in all three cases using alpha = 0 (ridge model).

Supplementary Table 1 Mass cytometry antibodies. Antigen, clone, label, vendor and catalog number, and staining concentration of extracellular antibodies (tab 1) and intracellular antibodies (tab 2) used for mass cytometry staining. *At time of rehydration, surface cocktail was supplemented with 8.64 µl of CD4 #201 (OKT4) per 12 LyoSpheres to increase signal.

Supplementary Table 2 Demographics of human donors. Age (years), height (cm), weight (kg), and BMI information of 39 females and 44 male human donors.

Supplementary Table 3 Stimuli. Stimuli marked with an asterisk (*) were dispensed at time of usage due to production and/or storage requirements.

Supplementary Table 4 Module assignments.

Supplementary Table 5 Reduced set of immune features. Suggestion for an immune monitory assay by traditional flow cytometry. The listed signaling proteins, cell types, and conditions provide surrogate markers for 10 of the 11 modules identified in our study and can be detected using an 8-parameter flow cytometry panel.

Supplementary Table 6 Selected features and coefficients for lasso models. Selected features and coefficients for lasso model of sex (tab 1), sparse group lasso model of sex (tab 2), and for lasso model of module scores to predict sex (tab 3).
