# Supplementary figures and images for: Variation of Immune Cell Responses in Humans Reveals Sex-Specific Coordinated Signaling Across Cell Types
Source: Front Immunol. 2022 Mar 28;13:867016. doi: 10.3389/fimmu.2022.867016 (PMC8995898; doi:10.3389/fimmu.2022.867016)

Figure S1:

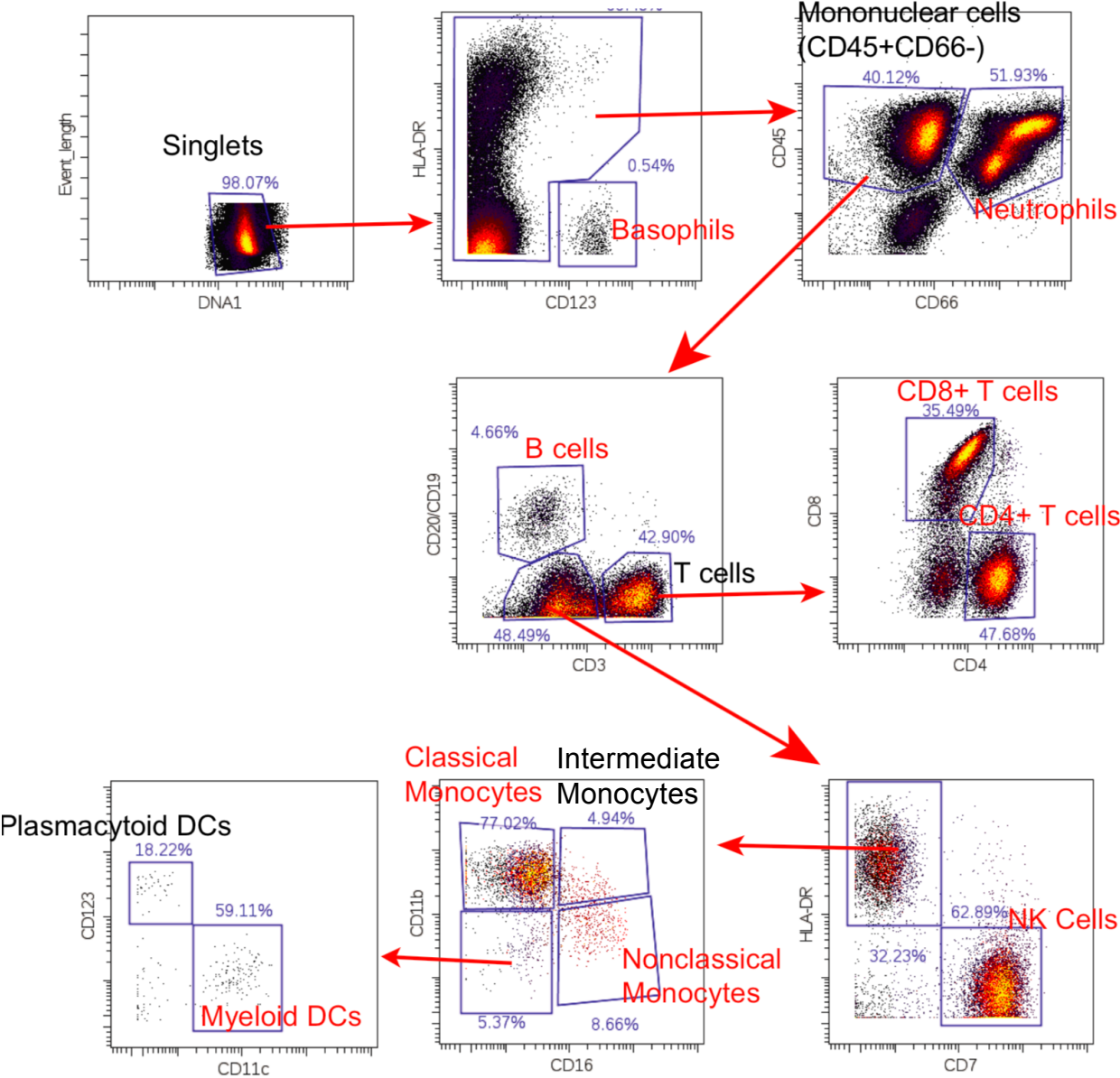

Supplement: Supplementary Figure 1 — Manual gating strategy for all data. Gating strategy identifying 12 unique cell populations for one representative donor. Populations used for analysis are shown in red. [file DataSheet_1.pdf]

Figure S3

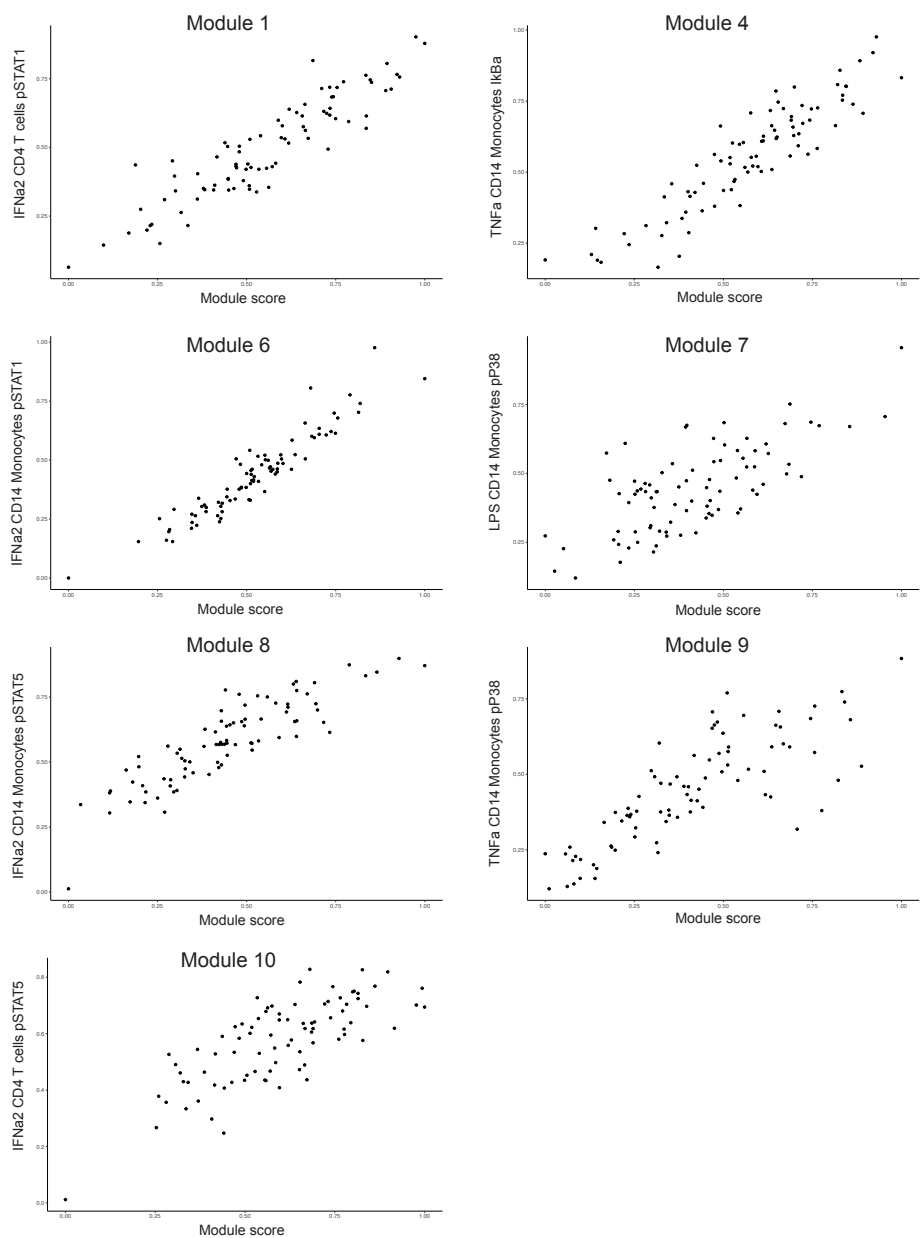

Supplement: Supplementary Figure 3 — Surrogate marker performance. Comparison of individual’s surrogate markers from a reduced feature set relative to their module scores using the full data set. Value on the y-axis is the value of the immune feature normalized to the range of that feature. Spearman rank correlations are as follows for modules 1, 4, 6, 7, 8, 9, 10: 0.90, 0.91, 0.94, 0.63, 0.88, 0.83, 0.74, respectively. [file DataSheet_3.pdf]

Figure S4

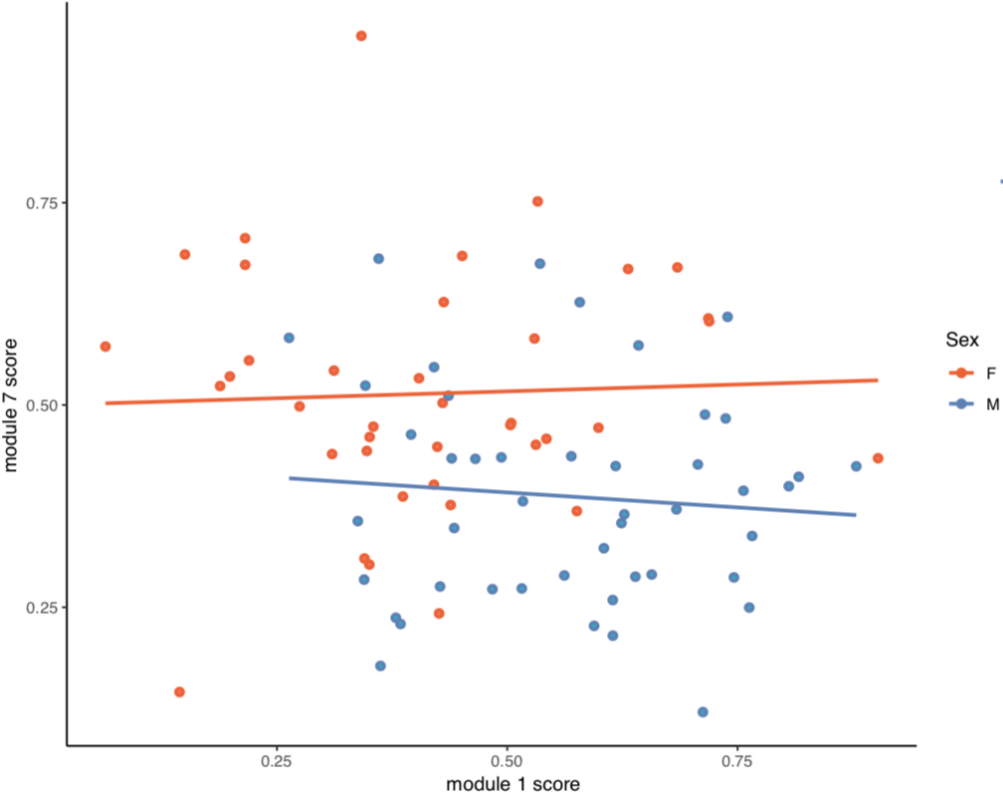

Supplement: Supplementary Figure 4 — Module 1 scores do not correlate with module 7 scores within the sexes. [file DataSheet_4.pdf]

Figure S5:

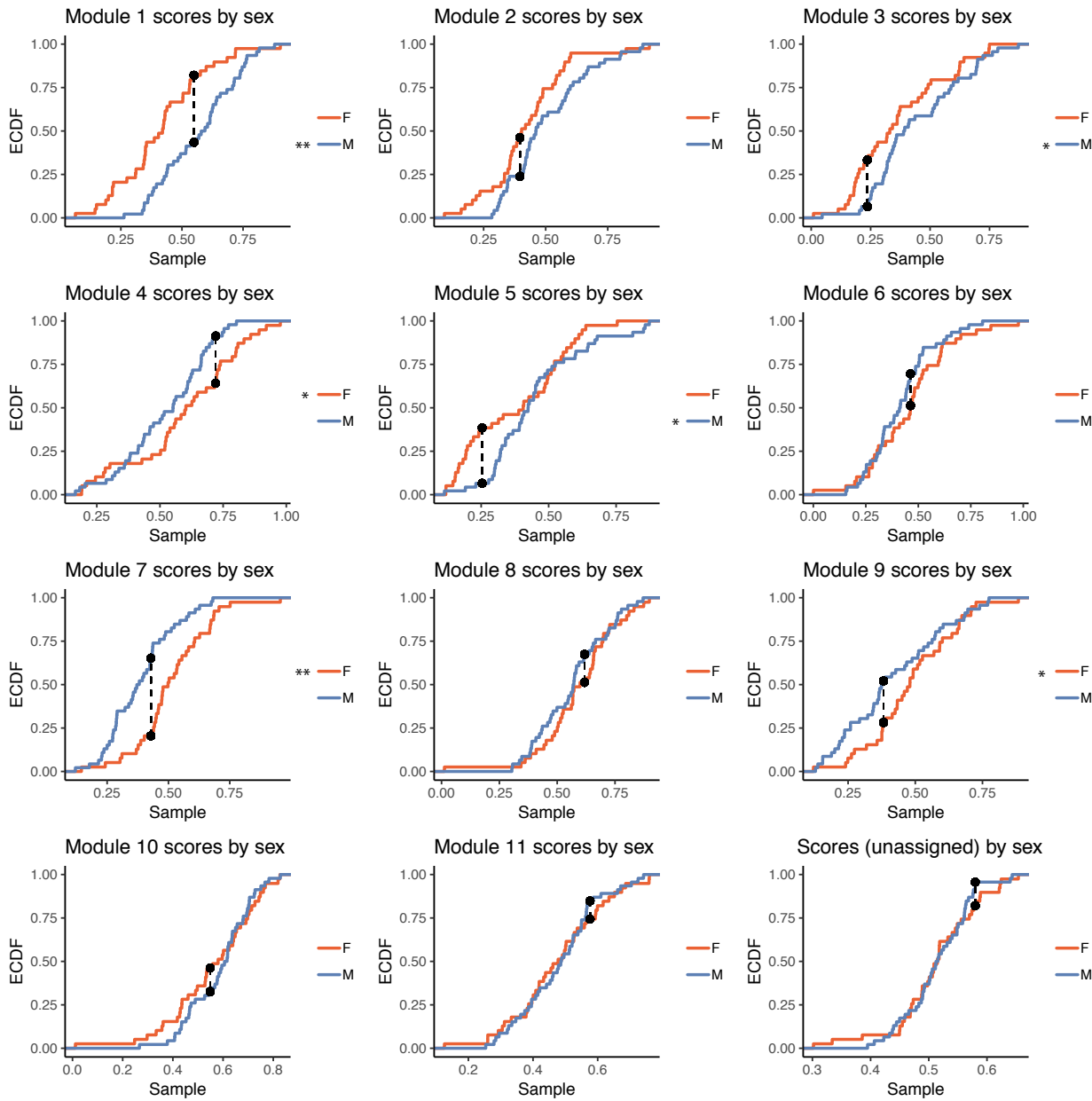

Supplement: Supplementary Figure 5 — Module score comparison between sexes. Comparison of module scores between sexes. Distributions marked with ** are significantly different when correcting for multiple hypothesis testing (Kolmogorov-Smirinov test, adjusted p-value < 0.05, h = 12). To show trends, all are modules are shown, those marked with * are significant without correcting for multiple hypothesis testing (Kolmogorov-Smirinov test, unadjusted p-value < 0.05). [file DataSheet_5.pdf]

Figure S6

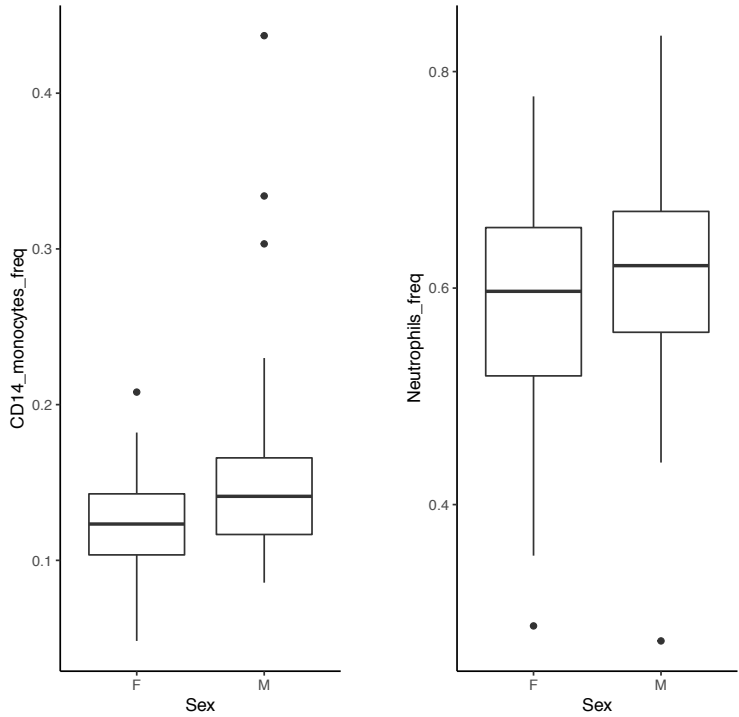

Supplement: Supplementary Figure 6 — Sex differences in frequency data. CD14+ monocyte frequencies (of mononuclear cells) and neutrophil frequencies (of singlets) in samples from male and female donors. Frequencies are higher in samples from male donors (Significance analysis of microarrays, FDR <.01). [file DataSheet_6.pdf]

Figure S7

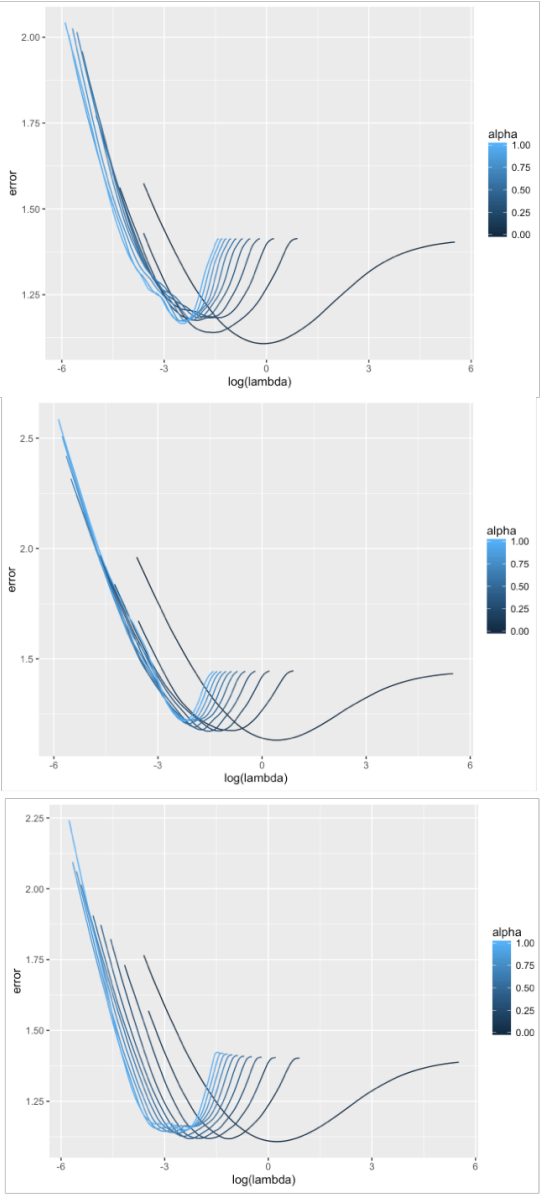

Supplement: Supplementary Figure 7 — Tuning alpha parameter for elastic net. Selection of alpha for elastic net modeling of sex differences. Three iterations of assigning k-groups for cross-validation are shown. Error is lowest in all three cases using alpha = 0 (ridge model). [file DataSheet_7.pdf]
