# Supplementary material for: Variation of Immune Cell Responses in Humans Reveals Sex-Specific Coordinated Signaling Across Cell Types
Source: Front Immunol. 2022 Mar 28;13:867016. doi: 10.3389/fimmu.2022.867016 (PMC8995898; doi:10.3389/fimmu.2022.867016)
Supplement: Supplementary Figure 2 — Boxplot of reference ranges of 199 immune features that exceeded a mean value of 0.2 arcsinh ratio. Immune features are grouped by cell type and colored by condition. [file DataSheet_2.pdf]

Figure S2:

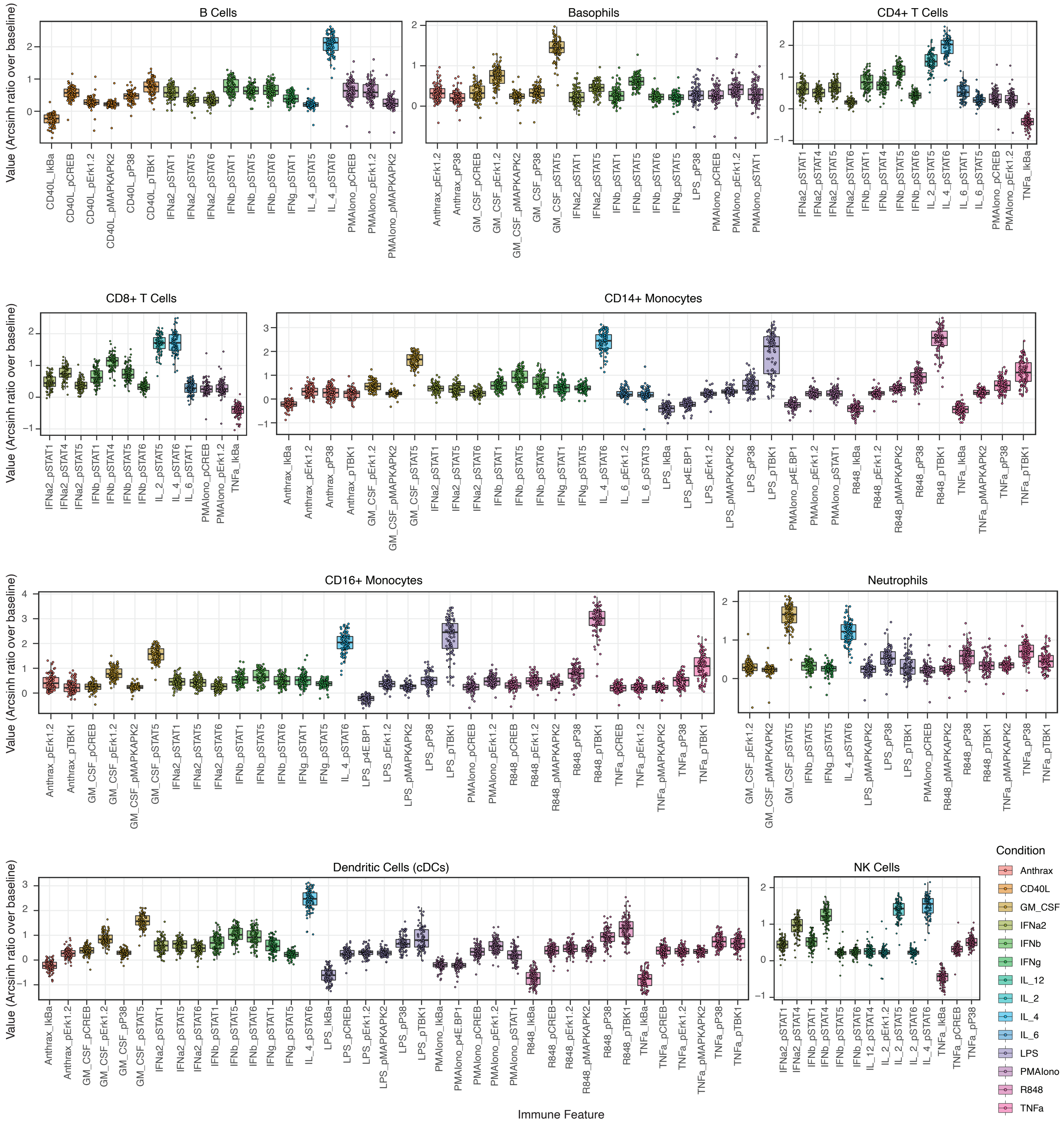
B Cells
CD40L_IkBa
CD40L_pCREB
CD40L_pErk1.2
CD40L_pMAPKAPK2
CD40L_pP38
CD40L_pTBK1
IFNa2_pSTAT1
IFNa2_pSTAT5
IFNa2_pSTAT6
IFNb_pSTAT1
IFNb_pSTAT5
IFNb_pSTAT6
IFNg_pSTAT1
IL_4_pSTAT5
IL_4_pSTAT6
PMAIono_pCREB
PMAIono_pErk1.2
PMAIono_pMAPKAPK2
Basophils
Anthrax_pErk1.2
Anthrax_pP38
GM_CSF_pCREB
GM_CSF_pErk1.2
GM_CSF_pMAPKAPK2
GM_CSF_pP38
GM_CSF_pSTAT5
IFNa2_pSTAT1
IFNa2_pSTAT5
IFNb_pSTAT1
IFNb_pSTAT5
IFNb_pSTAT6
IFNg_pSTAT5
LPS_pP38
PMAIono_pCREB
PMAIono_pErk1.2
PMAIono_pSTAT1
CD4+ T Cells
IFNa2_pSTAT1
IFNa2_pSTAT4
IFNa2_pSTAT5
IFNa2_pSTAT6
IFNb_pSTAT1
IFNb_pSTAT4
IFNb_pSTAT5
IFNb_pSTAT6
IL_2_pSTAT5
IL_4_pSTAT6
IL_6_pSTAT1
IL_6_pSTAT5
PMAIono_pCREB
PMAIono_pErk1.2
TNFa_IkBa
CD8+ T Cells
IFNa2_pSTAT1
IFNa2_pSTAT4
IFNa2_pSTAT5
IFNb_pSTAT1
IFNb_pSTAT4
IFNb_pSTAT5
IFNb_pSTAT6
IL_2_pSTAT5
IL_4_pSTAT6
IL_6_pSTAT1
PMAIono_pCREB
PMAIono_pErk1.2
TNFa_IkBa
CD14+ Monocytes
Anthrax_IkBa
Anthrax_pErk1.2
Anthrax_pP38
Anthrax_pTBK1
GM_CSF_pErk1.2
GM_CSF_pMAPKAPK2
GM_CSF_pSTAT5
IFNa2_pSTAT1
IFNa2_pSTAT5
IFNa2_pSTAT6
IFNb_pSTAT1
IFNb_pSTAT5
IFNb_pSTAT6
IFNg_pSTAT1
IFNg_pSTAT5
IL_4_pSTAT6
IL_6_pErk1.2
IL_6_pSTAT3
LPS_IkBa
LPS_p4E.BP1
LPS_pErk1.2
LPS_pMAPKAPK2
LPS_pP38
LPS_pTBK1
PMAIono_p4E.BP1
PMAIono_pErk1.2
PMAIono_pSTAT1
R848_IkBa
R848_pErk1.2
R848_pMAPKAPK2
R848_pP38
R848_pTBK1
TNFa_IkBa
TNFa_pMAPKAPK2
TNFa_pP38
TNFa_pTBK1
CD16+ Monocytes
Anthrax_pErk1.2
Anthrax_pTBK1
GM_CSF_pCREB
GM_CSF_pErk1.2
GM_CSF_pMAPKAPK2
GM_CSF_pSTAT5
IFNa2_pSTAT1
IFNa2_pSTAT5
IFNa2_pSTAT6
IFNb_pSTAT1
IFNb_pSTAT5
IFNb_pSTAT6
IFNg_pSTAT1
IFNg_pSTAT5
IL_4_pSTAT6
LPS_p4E.BP1
LPS_pErk1.2
LPS_pMAPKAPK2
LPS_pP38
LPS_pTBK1
PMAIono_pCREB
PMAIono_pErk1.2
R848_pCREB
R848_pErk1.2
R848_pMAPKAPK2
R848_pP38
R848_pTBK1
TNFa_pCREB
TNFa_pErk1.2
TNFa_pMAPKAPK2
TNFa_pP38
TNFa_pTBK1
Neutrophils
GM_CSF_pErk1.2
GM_CSF_pMAPKAPK2
GM_CSF_pSTAT5
IFNb_pSTAT5
IFNg_pSTAT5
IL_4_pSTAT6
LPS_pMAPKAPK2
LPS_pP38
LPS_pTBK1
PMAIono_pCREB
R848_pMAPKAPK2
R848_pP38
R848_pTBK1
TNFa_pMAPKAPK2
TNFa_pP38
TNFa_pTBK1
Dendritic Cells (cDCs)
Anthrax_IkBa
Anthrax_pErk1.2
GM_CSF_pCREB
GM_CSF_pErk1.2
GM_CSF_pP38
GM_CSF_pSTAT5
IFNa2_pSTAT1
IFNa2_pSTAT5
IFNa2_pSTAT6
IFNb_pSTAT1
IFNb_pSTAT5
IFNb_pSTAT6
IFNg_pSTAT1
IFNg_pSTAT5
IL_4_pSTAT6
LPS_IkBa
LPS_pCREB
LPS_pErk1.2
LPS_pMAPKAPK2
LPS_pP38
LPS_pTBK1
PMAIono_IkBa
PMAIono_p4E.BP1
PMAIono_pCREB
PMAIono_pErk1.2
PMAIono_pSTAT1
R848_IkBa
R848_pCREB
R848_pErk1.2
R848_pMAPKAPK2
R848_pP38
R848_pTBK1
TNFa_IkBa
TNFa_pCREB
TNFa_pErk1.2
TNFa_pMAPKAPK2
TNFa_pP38
TNFa_pTBK1
NK Cells
IFNa2_pSTAT1
IFNa2_pSTAT4
IFNb_pSTAT1
IFNb_pSTAT4
IFNb_pSTAT5
IFNb_pSTAT6
IL_12_pSTAT4
IL_2_pErk1.2
IL_2_pSTAT5
IL_2_pSTAT6
IL_4_pSTAT6
TNFa_IkBa
TNFa_pCREB
TNFa_pTBK1
Value (Arcsinh ratio over baseline)
Immune Feature
Condition
Anthrax
CD40L
GM_CSF
IFNa2
IFNb
IFNg
IL_12
IL_2
IL_4
IL_6
LPS
PMAIono
R848
TNFa
