# Supplementary material for: Variation of Immune Cell Responses in Humans Reveals Sex-Specific Coordinated Signaling Across Cell Types
Source: Front Immunol. 2022 Mar 28;13:867016. doi: 10.3389/fimmu.2022.867016 (PMC8995898; doi:10.3389/fimmu.2022.867016)
Supplement: Supplementary Table 3 — Stimuli. Stimuli marked with an asterisk (*) were dispensed at time of usage due to production and/or storage requirements. [file Table_3.docx]

| **Stimulus** | **Host Species** | **Produced In** | **Lot** | **Working Concentration** |
| --- | --- | --- | --- | --- |
| GM-CSF | Human | E. coli | 3112711 | 100 ng/ml |
| IFNα2 | Human | E. coli | 5962 | 150 ng/ml |
| LPS | N/A | E. coli O111:B4 | LEB-36-01 | 1 μg/ml |
| IL-6 | Human | E. coli | 3103810 | 500 ng/ml |
| Resiquimod (R848) | N/A | N/A | 848-35-14 | 10 μg/ml |
| IFNγ | Human | E. coli | RAX1814011 | 330 ng/ml |
| TNFα | Human | E. coli | DDHB0113062 | 100 ng/ml |
| IFNβ* | Human | CHO | 5886 | 5 ng/ml |
| CD40L soluble dimer (“MegaCD40L”) | Human | CHO | 05281412, 03041401 pooled | 125 ng/ml |
| PMA and ionomycin* | N/A | N/A | E13495-116 | 0.081 μM PMA and 1.34 μM iono |
| IL-12 | Human | CHO | 0210596, 0707596-2 pooled | 400 ng/ml |
| IL-4 | Human | E. coli | AG1314021 | 125 ng/ml |
| IL-2 | Human | E. coli | 101312, 041412 pooled | 2 μg/ml |
| *Gamma-inactivated vegetative *Bacillus anthracis* Ames | N/A |  | AGD0001331 | 400,000 CFU/ml |
| *Zaïre Ebolavirus-like particles | N/A | 293T cells |  | Varied |
