# Supplementary material for: Variation of Immune Cell Responses in Humans Reveals Sex-Specific Coordinated Signaling Across Cell Types
Source: Front Immunol. 2022 Mar 28;13:867016. doi: 10.3389/fimmu.2022.867016 (PMC8995898; doi:10.3389/fimmu.2022.867016)
Supplement: Supplementary Table 5 — Reduced set of immune features. Suggestion for an immune monitory assay by traditional flow cytometry. The listed signaling proteins, cell types, and conditions provide surrogate markers for 10 of the 11 modules identified in our study and can be detected using an 8-parameter flow cytometry panel. [file Table_5.docx]

| **Signaling proteins:** | **Conditions:** | **Proposed panel:** |
| --- | --- | --- |
| p-STAT1 | Unstimulated control | CD45 |
| p-STAT5 | TNFa | CD3 |
| IkB | IFNa | CD4 |
| p-P38 | LPS | CD14 |
|  |  | p-STAT1 |
| **Cell types:** |  | p-STAT5 |
| CD4+ T cells |  | IkB |
| CD14+ Monocytes |  | p-P38 |
